# Requirements for ethics committee review for studies submitted to *Implementation Science*

**DOI:** 10.1186/1748-5908-6-32

**Published:** 2011-03-31

**Authors:** Martin P Eccles, Charles Weijer, Brian Mittman

**Affiliations:** 1 Institute of Health & Society, Baddiley-Clark Building, Newcastle upon Tyne, NE2 4AX, UK; 2 Rotman Institute of Philosophy, University of Western Ontario, London, N6A 5B6, Canada; 3 VA Center for Implementation Practice and Research Support, VA Greater Los Angeles Healthcare System, 16111 Plummer Street, Sepulveda, CA 91343, USA

## Abstract

The requirement for ethics review of studies submitted to *Implementation Science *has been unclear. Therefore, in this editorial, we set out our requirements for ethics committee review of experimental and non-experimental studies. For any study that meets the criteria of human subject research (which includes research on healthcare providers), irrespective of study design, we will require proof of either satisfactory ethics committee review or of the granting of an official exemption or waiver.

## Background

The requirement for ethics committee review of studies submitted to *Implementation Science *is unclear. We regularly receive manuscripts that have not been reviewed by an ethics committee; there appear to be four general reasons for this. First, authors claim that studies with no research intervention do not need ethics committee approval. Second, authors from some countries (*e.g*., The Netherlands) say that their system exempts certain types of study from the need for ethics committee review and so they have not, and do not see the need to, approach an ethics committee. However, we have seen a clear dichotomy in practice within such countries, with some authors choosing to approach ethics committees and some not. Third, authors claim that what they have done is 'quality improvement' and so is exempt from the need for ethics review. Finally, particularly in international studies, authors may be unclear about how and where to obtain appropriate ethics review.

Our instructions to authors clearly state the need for ethics committee review of randomised controlled trials and other studies that involve the delivery of a research intervention. However, our requirements in relation to non-intervention research studies are not stated. Therefore, to clarify the position for authors considering submission to *Implementation Science*, we set out in this editorial the key considerations that guide our judgements on the need for ethics committee review and what we require of authors.

## The need for ethics committee review

The *Declaration of Helsinki *requires that all medical research be submitted to and approved by an ethics committee. It states: 'The research protocol must be submitted for consideration, comment, guidance and approval to a research ethics committee before the study begins' [1]. It is well understood that legal and ethical requirements for research vary from one country to another. Thus, in its review, an ethics committee will 'take into consideration the laws and regulations of the country or countries in which the research is to be performed as well as applicable international norms and standards but these must not be allowed to reduce or eliminate any of the protections for research subjects set forth in this Declaration' [1]. Manuscripts submitted to *Implementation Science *must meet these international ethical requirements.

Why the requirement for ethics review? The answer is straightforward: because research involving human subjects puts people at risk. The risks may be physical, psychological, social, economic, legal, or dignitary; a single study may also pose more than one type of risk to subjects. Of course, people are exposed to risks all the time, whether it is at work, in the doctor's office, or driving a car. The difference in research is that people are exposed to risk in large part for the benefit of others, be they other patients, the health system, or society at large. It is this feature of research that drives the need for independent ethics review. While the integrity of researchers remains an important protection for research subjects, researchers themselves may not be in a good position to make the best judgement regarding the ethical acceptability of a research study. Ethics committees ensure that the liberty and welfare interests of research subjects are protected, and that national and international ethical and legal requirements are upheld.

We understand the *Declaration of Helsinki *to require that all research involving human subjects be submitted to an ethics committee for review and approval, or to be determined exempt from the need for review. Unfortunately, the *Declaration of Helsinki *leaves some key terms undefined. Research may be usefully defined as 'a class of activities designed to develop or contribute to generalizable knowledge' ([2] p. 3). A human subject is 'a living individual about whom an investigator (whether professional or student) obtains (1) data through intervention or interaction with the individual, or (2) identifiable private information' ([2] p. 8). Therefore, any study that involves intervening upon people (*e.g*., a complex intervention involving educational sessions), interacting with them (*e.g*., conducting an interview or administering a questionnaire), or collecting identifiable private data (*e.g*., abstracting data with identifiers from a medical record) for the purpose of contributing to generalizable knowledge must be submitted to an ethics committee. This applies to studies involving healthcare providers as research subjects just as much as it does to studies involving patients. Therefore studies (with or without a research intervention) require ethics review if they involve interaction with human subjects or the collection of identifiable private information. Research using social, behavioural, or economic research methods may not include a research intervention, but such research exposes human subjects to risk nonetheless. Studies in which people are interviewed may pose psychological risks by asking sensitive questions or risks resulting from a breach of confidentiality. When researchers only collect identifiable information from patient medical records risks from a breach of confidentiality remain. The authors of *Protecting Participants and Facilitating Social and Behavioral Sciences Research *argue that while these risks in social, behavioural, or economic studies necessitate ethics committee oversight, it should be 'appropriately tailored to risk' [3].

## What do we expect of ethics committees?

We believe that the conduct of research on human subjects requires an independent judgement by a legally constituted ethics committee; this is not a judgement that can safely be made by researchers. We expect ethics committees to make a disinterested decision, guided by the type of study, on the balance of benefits and harms to study research subjects, the need for informed consent, and the need for other protections. It is likely that many non-intervention studies will confer only small benefits to research subjects and carry small risk of harm, with the most likely harm resulting from disclosure of personal information. In such 'low risk' studies we would hope that ethics review committees would have an expedited process for considering them so as not to place a disproportionate burden on the investigators nor consume a disproportionate proportion of an ethics committee's limited resources.

## Ethics committee review and the requirement for informed consent

The requirement for ethics committee review and the requirement for informed consent are often conflated. Does our requirement for ethics committee review mean that researchers must automatically always be obtaining informed consent from research subjects? No. While there is a general presumption that informed consent will be obtained in research, the requirements for ethics review and informed consent are separable. In many jurisdictions, researchers may request a waiver of the requirement for informed consent from an ethics committee when they submit a study for review. For example, the CIOMS *International Ethics Guidelines *state that 'when the research design involves no more than minimal risk and a requirement of individual informed consent would make the conduct of the research impracticable ... the ethical review committee may waive some or all of the elements of informed consent' [4]. A waiver of (or an exemption from the need for) consent may be applied when a study only involves recording data from the medical record or administering a questionnaire that poses low risk.

## What type of study does not require ethics committee review?

Of the types of study that we regularly publish neither systematic reviews nor debate articles require ethics committee review. Secondary analyses of suitably anonymised datasets would not require ethics committee review either, but authors of such studies would need to clearly describe the situation with regard to ethical considerations within the methods section of their manuscript. Most other types of study will require ethics committee review as described below; this includes both completed studies and study protocols.

## Does quality improvement need ethics review?

Some authors will describe their work as 'quality improvement' (or some such term denoting service development activities rather than research), which would not require ethics committee review. If such studies are genuinely service development then we agree that they do not need ethics committee review but we do not consider them as meeting our definition of research as articulated above. Therefore, such articles are not within the scope of *Implementation Science *and we would not wish to publish them. Should authors have used a label of quality improvement as a way of side-stepping (the perceived bureaucratic burden of) ethics committee review but subsequently wish their work to be considered for publication as research then we will require them to submit it for (retrospective) ethics committee review.

## What we require of authors

In all manuscripts (of either completed studies or study protocols) describing research on human subjects (irrespective of study design), we require authors to provide the following information.

In the case of studies that have been reviewed by an ethics review committee, we require an explicit statement at the end of the Methods section stating that the study has been approved by a legally constituted ethics committee and giving the name and study reference of the committee. Study protocols must be accompanied by a copy of the approval letter submitted along with the manuscript.

In the case of studies that have been exempted from review, we require an explicit statement at the end of the Methods section stating that the study has been submitted to a legally constituted ethics committee and deemed exempt from review and giving the name and study reference of the committee. If this process has not been conducted by an ethics committee, then we require a copy of the letter detailing the permissions; depending on the provenance of the letter, we may need to ask for further clarification. If we judge that the author of the letter did not appropriately fulfil the role of an ethics committee, then we will require the author to submit the study to a legally constituted ethics committee. In such cases, we will not take any further decisions until either the study is approved or deemed exempt from review.

If a study has not been submitted to a legally constituted ethics committee, then we will require authors to submit it and will not take any further decisions until either it is approved or the committee deems it exempt from review. The subsequent decision should be recorded in an explicit statement at the end of the Methods section stating that the study has been submitted to a legally constituted ethics committee, report the committee name, the decision, and study reference of the committee.

It is important that authors understand that this process occurs as part of *Implementation Sciences' *initial manuscript checks and before an editorial decision is made regarding the suitability of an article for consideration by the journal.

## Competing interests

The authors declare that they have no competing interests.

## Authors' contributions

MPE conceived of the idea for the article. All authors contributed ideas and comments into a first draft. The writing was led by MPE and CW. All authors approved the final draft.
